# Long term results of valve-sparing aortic root reconstruction

**DOI:** 10.1186/1749-8090-8-S1-O49

**Published:** 2013-09-11

**Authors:** SJ Oh, KH Lee, HY Hwang, KH Kim, KB Kim, H Ahn

**Affiliations:** 1 Department of Thoracic and Cardiovascular Surgery, SMG-SNU Boramae Medical Center, Seoul, Korea

## Background

To present the early and late clinical outcomes of valve-sparing aortic root reconstruction in patients with the various types of aortic root disease.

## Methods

Between June 1996 and November 2012, 21 patients who underwent aortic valve sparing for aortic root aneurysm were retrospectively reviewed. Mean age was 45 ± 16 years. 9 patients had Marfan syndrome and one patient had Takayasu arteritis. All patients had tricuspid aortic valve except one patient with bicuspid valve, and 15 patients showed moderate or severe degree of aortic regurgitation. 15 patients were diagnosed with annuloaortic ectasia, 2 with ascending aortic aneurysm, 2 with aortic dissection and 2 with sinus valsalva aneurysm. Reimplantation of aortic root was performed in 13 patients and remodeling techniques in 8. Mean follow up duration was 69.4 months.

## Results

There were no operative mortality and one late-death. Survival of total patients at 5, 10, 15 years were 100, 83.3, 83.3%, respectively. There was no major complication related to the operation. Moderate aortic regurgitation developed in 6 patients, but there was no severe aortic regurgitation. Any patient did not require reoperation during the follow up periods. Freedom from moderate or severe aortic regurgitation at 5 and 10 years were 82.5 ± 9.3 % and 70.7 ± 13.5 % for all patients, and there was no significant difference according to the type of operation. (80.8 ± 12.6%, reimplantation vs. 71.4 ± 17.1%, remodeling, p=0.440).

## Conclusions

Aortic valve-sparing aortic root reconstruction including both implantation and remodeling technique provides the good clinical outcomes and is associated with low rates of valve related complications.

